# Surviving and fatal Elephant Endotheliotropic Herpesvirus-1A infections in juvenile Asian elephants – lessons learned and recommendations on anti-herpesviral therapy

**DOI:** 10.1186/s12917-016-0806-5

**Published:** 2016-08-27

**Authors:** Akbar Dastjerdi, Katharina Seilern-Moy, Karin Darpel, Falko Steinbach, Fieke Molenaar

**Affiliations:** 1 Animal and Plant Health Agency-Weybridge, New Haw, Addlestone, Surrey, KT15 3NB UK; 2 School of Veterinary Medicine, Faculty of Health and Medical Sciences, University of Surrey, Guildford, GU2 7XH UK; 3 ZSL Whipsnade Zoo, Dunstable, Bedfordshire LU6 2LF UK

**Keywords:** EEHV-1, elephantid herpesvirus, EEHV-haemorrhagic disease, EEHV-HD, famciclovir, ganciclovir

## Abstract

**Background:**

Elephant Endotheliotropic Herpesviruses (EEHVs) can cause acute haemorrhagic disease in young Asian elephants (*Elephas maximus)* and clinical EEHV infections account for the majority of their fatalities. The anti-herpesviral drug famciclovir (FCV) has been used routinely to treat viraemic at-risk elephants, but thus far without proven efficacy. This paper presents clinical and virological investigations of two EEHV-1A infected elephants treated with FCV, and discusses anti-herpesvirus therapies of viraemic elephants.

**Cases presentations:**

Two 1.5 year old male Asian elephants at a zoological collection in the UK developed clinical EEHV-1A infections. Case 1 showed signs of myalgia for the duration of 24 hours before returning back to normal. EEHV-1A DNAemia was confirmed on the day of clinical signs and continued to be present for 18 days in total. Trunk shedding of the virus commenced 10 days after detection of initial DNAemia. Case 2 tested positive for EEHV-1A DNAemia in a routine blood screening sample in the absence of clinical signs. The blood viral load increased exponentially leading up to fatal clinical disease seven days after initial detection of DNAemia. Both calves were treated with 15 mg/kg FCV per rectum on detection of DNAemia and penciclovir, the FCV metabolite, could be detected in the blood at assumed therapeutic levels. The early indicators for clinical disease were a marked absolute and relative drop in white blood cells, particularly monocytes prior to the detection of viraemia. The most prognostic haematological parameter at later stages of the disease was the platelet count showing a continuous sharp decline throughout, followed by a dramatic drop at the time of death.

**Conclusions:**

The EEHV-1A viraemic animals investigated here further highlight the ongoing threat posed by these viruses to juvenile Asian elephants. The findings call into question the efficacy of rectal FCV in clinical cases and direct towards the use of alternative anti-herpesvirus drugs and complementary treatments such as plasma infusions if no improvement in either viral load or the above-mentioned blood parameters are observed in the initial days of viraemia despite anti-herpesvirus therapy.

## Background

Elephant Endotheliotropic Herpesviruses (EEHVs) or Elephantid Herpesviruses (ElHVs) belong to the genus *Proboscivirus* of the family *Herpesviridae* [1, 2] and fall into 7 genotypes [3–9]. Phylogenetically, the viruses are most closely, although distantly, related to human cytomegalovirus (HCMV) and three human *Roseolo* viruses (human herpesvirus-6A, -6B, and -7), all members of the subfamily *Betaherpesvirinae* [1].

EEHVs, in particular EEHV-1, are the cause of acute haemorrhagic disease (EEHV-HD) in young Asian elephants (*Elephas maximus*), and clinical EEHV infections account for most of the fatalities in juvenile Asian elephants, both in their natural habitat and in captivity [7–13]. EEHV-1 induced acute haemorrhagic disease is manifested initially by sudden onset of non-specific clinical signs, which often develop into haemorrhagic disease and sudden death within 1 to 7 days [9, 12]. It has also been documented that EEHV viraemia is detectable at least several days before the onset of clinical signs [13, 14]. To enable early therapeutic intervention in response to detection of EEHV viraemia, some zoological collections have implemented regular blood sampling and laboratory testing of juvenile Asian elephants.

Conferring from herpesviruses’ properties, it could be assumed that once an elephant is infected it remains a latent carrier for life. Screenings of elephant herds have revealed widespread prevalence of the viruses and their periodical reactivation and shedding through biological fluids, especially trunk secretions [13–19]. Routes of EEHVs transmission to naïve individuals are still unclear, however, considering other herpesviruses’ transmission routes, contact with infected bodily secretions is a very likely possibility and vertical transmission cannot be excluded.

EEHVs, similar to viruses in the subfamily *Alphaherpesvirinae* and *Gammaherpesvirinae*, comprise of a genome with a thymidine kinase (TK) and a serine/threonine protein kinase (PK) [7, 20], which encode enzymes crucial for processing many anti-herpesviral prodrugs into their active derivatives. Based on approaches in treatment of other herpesviruses with TK and PK genes, three nucleoside analogues [famciclovir (FCV) and more recently ganciclovir (GCV) and aciclovir (ACV)] have been administered to treat viraemic elephant calves, with the aim to avert progression to clinical disease, but as yet without proven clinical efficacy. It is known that several elephant calves have survived laboratory confirmed EEHV infections. These elephant calves were treated with FCV at doses of 5.5-8.0 mg/kg twice daily (BID) rectally [9, 18, 19, 21–24] or with GCV at 5 mg/kg BID intravenously followed by oral administration [14, 25]. One elephant calf with advanced EEHV-1 clinical signs has been treated successfully with ACV at 12mg/kg BID intravenously for 15 days along with penicillin G and supportive treatment including vitamin C, physiological saline and 5 % Dextrose [26]. Apart from the latter case, it seems likely that many of surviving elephant calves represent relatively mild cases, in view of length of time taken to reach the highest viral load, level of viraemia at this point and recovery of blood parameters. It is therefore likely that these affected individuals might have recovered without drug intervention [9].

FCV, chemically known as 2-[2-(2-amino-9H-purin-9-yl)ethyl]-1,3-propanediol diacetate, is a synthetic acyclic guanosine analogue that is rapidly de-acetylated and oxidized in the body to its metabolite penciclovir (PCV). Activation of PCV requires phosphorylation in the virally infected cells by viral and cellular kinases. The drug has a half-life of approximately three hours and its catabolite is excreted renally [27]. PCV interacts with herpesvirus DNA polymerase, the enzyme responsible for creating DNA molecules through assembly of nucleotides (building blocks of DNA), and thus terminates DNA synthesis. A recent study showed that when administered to healthy young Asian elephants both orally or per rectum, FCV is absorbed and metabolised to PCV with plasma levels considered therapeutic in humans [27, 28]. ACV, another guanosine analogue with a biological half-life of 2-4 hours, is still used widely for the treatment and prevention of clinical Herpes Simplex Virus (HSV) and Varicella Zoster Virus (VZV) diseases in humans. Activation of ACV within cells via the viral and cellular kinases yields ACV–triphosphate, which interacts adversely with herpesvirus DNA synthesis [29]. GCV is another guanosine analogue that is activated via viral and cellular kinases. Its oral prodrug valganciclovir (VGCV) is the most commonly prescribed drug for the prevention and therapy of HCMV in immunocompromised patients [29].

This paper describes the investigations into two EEHV-1A viraemic elephants from a UK zoological collection that were both treated with FCV. It also discusses findings of the investigations in relation to anti-herpes viral therapies in EEHV viraemic elephants.

## Case presentations

### Virus screening

Blood samples of EDTA whole blood and blood swabs were collected under trained behaviour from an ear vein after aseptic preparation of the medial aspect of the ear pinna. For EDTA whole blood samples, the animal was placed in lateral recumbency and the lowermost ear was sampled, using a standard 21G 1-inch needle and a Vacutainer® blood collection system. For blood swabs, a dry 6-inch foam tipped swab (Sigma Aldrich, UK) was used for collection of a drop of blood after puncture of an ear vein with an insulin needle, with the elephant in standing position. Blood samples were kept at room temperature before being shipped to the laboratory, where they were stored at 4–8 °C until being processed. Under trained behaviour, conjunctival swabs and trunk swabs were collected. Conjunctival swabs were collected using a sterile dry swab for each eye, running these on the inside of the lower palpebra. Trunk secretions from the mucosa of each naris were collected on a dry 6-inch foam tipped swab at a depth of approximately 8–10 cm, under trained behaviour, and stored at −20 °C prior to further testing. Blood smears were made of freshly collected EDTA blood samples, air dried and stained with Diff-Quick (LabPak, UK) for an in-house relative white blood cell (WBC) differential count.

Nucleic acid was extracted from 140 μl EDTA whole blood using the QIAamp® Viral RNA Mini kit (Qiagen, UK) according to the manufacturer’s protocol. For blood swabs, 560 μl buffer AVL from the kit were directly added to the foam tipped swabs, left for 15 minutes at room temperature, vortexed and spun briefly, and processed as per the manufacturer’s instructions. To process trunk swab samples for nucleic acid extraction, 300 μl PBS and 6–8 glass beads (3 mm in diameter) were added to the swab inside a 2 ml Eppendorf tube, vortexed briefly and incubated for 30 minutes at room temperature. The tube content was then vortexed and nucleic acid was extracted following the EDTA whole blood protocol using 140 μl of the cleared suspension.

The qPCR used in this study was performed as described [16] using the QuantiFast® Pathogen PCR + IC kit (Qiagen). Briefly, 2 μl extracted DNA was added to 0.1 μl each forward and reverse primer (100 pmol/μl), 0.05 μl hydrolysis probe (100 pmol/μl), 5 μl 5× QuantiFast Pathogen Master Mix, 0.5 μl 50× High-ROX Dye solution, and 17.25 μl RNase-Free Water. The qPCR was performed on a MX3005P machine (Agilent Technologies, UK) using the cycling conditions of one cycle of 95 °C for five minutes followed by 45 cycles of 95 °C for 15 seconds, 60 °C for 30 seconds, and signal acquisition at the end of each cycle. The conventional PCRs were carried out as described [30] using several primer pairs to amplify partial sequences of polymerase (POL), glycoprotein L (gL) and gM genes.

The DNA standard used was a HPLC purified 100 bases synthetic oligonucleotide (Eurofins Genomics, UK), identical to the sequence of the qPCR target, diluted to provide serial copy numbers from 10^6^ to 10^1^ copies per PCR test. The synthetic oligonucleotide dilutions were run in triplicate in the PCRs to create a standard curve for EEHV genome quantification.

#### Supportive therapy and drug administration

Rectal fluids were provided after manual removal of all faeces from the rectum. Several litres of hand-warm water were then deposited into the distal colon at approximately one meter depth, through a standard garden hose pipe. Intravenous (i/v) access was acquired after antiseptic preparation of the medial aspect of the ear pinna, using an 18G 2.5-inch indwelling catheter (Milacath®, Mila International, USA) in an ear vein. The catheter was secured using Durapore® tape and superglue. Standard giving sets were used for i/v fluids and filtered giving sets for the administration of blood and blood components (Baxter, UK). Plasma was collected from adult herdmates under trained behaviour, in lateral recumbency from either an ear vein or a medial hind leg vein, in one litre citrate blood collection bags (Crusse, Denmark), stored for a minimum of eight hours at 5 °C and separated using manual pressure. The plasma was then stored at 5 °C and warmed to room temperature prior to administration.

Anti-herpes viral treatment consisted of either FCV or GCV. FCV (Novartis Pharmaceuticals, UK) 250 and/or 500 mg tablets were crushed using a pestle and mortar and suspended in hand warm water. Using a 60 ml syringe and a meter long tube, the suspension was administered in the distal colon after removal of all faeces as described above. GCV (Cymevene® 500 mg, Roche, UK) was diluted in one litre of physiological saline attached to a giving set and an i/v catheter, and administered slowly over a period of one hour.

For measurement of plasma levels of GCV and PCV, the FCV metabolite, whole blood samples obtained approximately 6-10 hours following drugs administration, were centrifuged at 800 × g for 10 minutes and plasma was collected and stored at −20 °C for later analysis. Plasma samples were analysed at the College of Veterinary Medicine, University of Tennessee, USA using a reverse phase HPLC method with solid phase extraction (SPE). The compounds were separated on an Atlantis T3 (4.6 × 250 mm) column with a mobile phase of 10 mM ammonium phosphate pH 2.9 and acetonitrile (95:5). The flow rate was 1.2 ml/min and fluorescence was measured at excitation 253 nm and emission 360 nm. Standard curves for plasma analysis were prepared by fortifying untreated, pooled elephant plasma with the drug to produce a linear concentration range of 10-5000 ng/ml. Average recovery was 100 % while intra and inter-assay variability ranged from 0.5 to 5.1 %. The lower limit of quantification was 10 ng/ml.

### Case 1

**Fig. 1:**
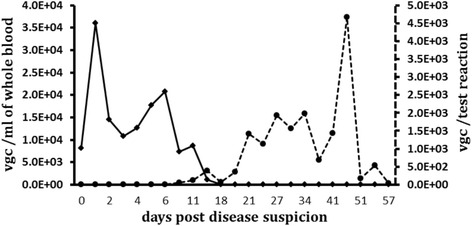
EEHV-1A DNA levels in blood and trunk secretions of Case 1. Clinical suspicion of EEHV infection was confirmed by detection of EEHV-1A DNA in blood. The animal showed clinical signs suggestive of active EEHV-1A infection on Day 0 and Day 1. EEHV-1A DNA load in EDTA whole blood (solid line) and trunk secretions (dashed line) was measured using a qPCR

**Fig. 2:**
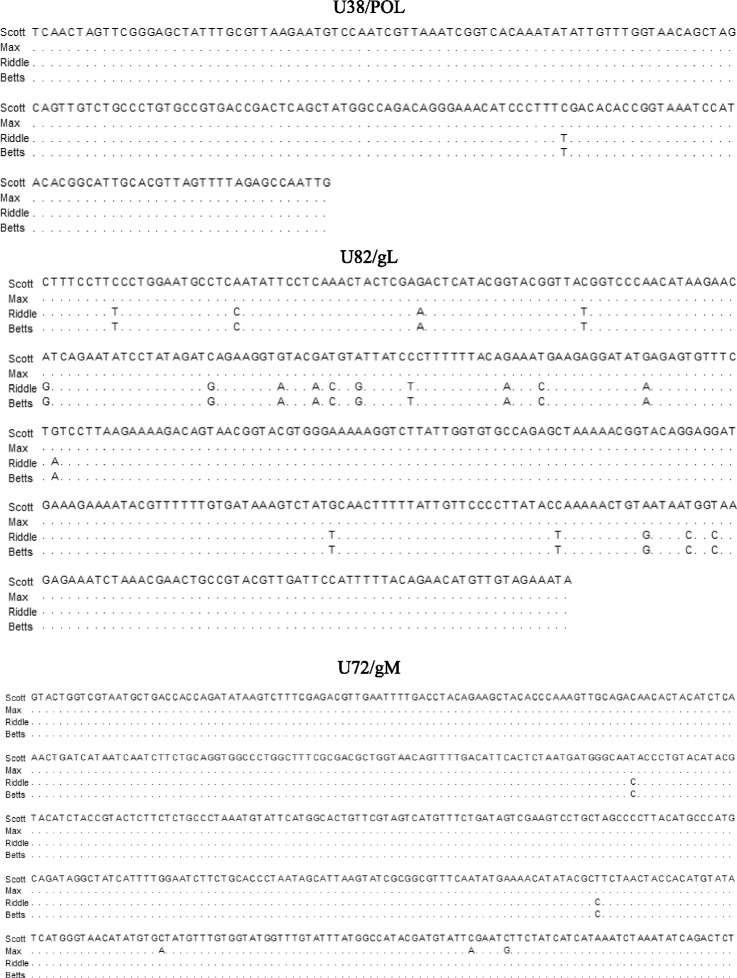
Nucleotide sequence comparison of the EEHV-1A detected. Partial nucleotide sequences of DNA polymerase [U38/POL (194 bp)], glycoprotein L [U82/gL (381 bp)] and glycoprotein M [U72/gM (500 bp)] from Case 1 and Case 2 were compared with those of two previous EEHV-1A fatalities (Riddle and Betts) from the same zoological collection. Sequences were aligned using the MegAlign 13 software of the DNASTAR Lasergene 13 package (DNASTAR Inc. Madison, USA). Identical nucleotides to those of Case 1 are denoted by dots in the alignments

In 2013, 1.5 year old male Asian elephant calf ‘Scott’ was presented with unwillingness to bend either foreleg, swinging both legs forward when walking. Its ears were drooping, the head was down and the calf was off solid food. An EDTA whole blood sample taken on the day tested qPCR positive for EEHV-1 DNA. Prior to this event, routine blood samples and trunk swabs tested negative for EEHV-1. Using a qPCR assay, the blood viral load was determined as 8.16*10^3^ viral genome copies (vgc)/ml (Fig. 1). Genomic analysis of this EEHV-1 by sequencing of PCR amplicons of partial viral POL, gL and gM genes identified the virus as EEHV-1A genotype. The sequences comparison with those of two EEHV-1A fatalities in 2009 (Riddle and Betts) at the collection revealed several nucleotide substitutions in the POL, gL and gM gene segments, indicating a genomically different virus from those in Riddle and Betts (Fig. 2).

Treatment with 15 mg/kg FCV three times daily (TID) per rectum was instigated eight hours after these initial clinical signs. The first two treatments, six hours apart, consisted of ground FCV tablets suspended in ultrasound gel (Healthlife®, Barclays-Swan Ltd, UK); however, most of the drug was expelled at the next defaecation. Twelve hours after the initial presentation, the calf started to voluntarily bend his right foreleg again, and by the next morning, despite some stiffness in the shoulders, the legs were in normal use and its appetite had returned. The dose of FCV was therefore reduced to 12 mg/kg TID (Day 1); this time dissolved in hand warm water and placed 1.5 meters deep into the rectum. All clinical signs were resolved by Day 2, when the blood viral load peaked at 3.61*10^4^ vgc/ml, after which it started to decrease gradually. The FCV dose was further reduced to 8 mg/kg BID on Day 7. Full haematology at this point showed a relatively low monocyte count (5.94*10^9^/L, 26 % of WBC), but was normal (51 % of WBC) when tested three weeks later (Table 1a). No virus was detectable in the blood from Day 18 onwards (Fig. 1), and FCV treatment ceased on Day 21. Viral DNA excretion through eye (data not shown) and trunk secretions commenced on Day 10 and continued for at least two months, with a peak at around Day 48 of 4.66*10^3^ vgc/test reaction. No further samples were tested beyond Day 57.

**Table 1:** Haematological parameters of Case 1 and Case 2 measured during clinical EEHV-1A infection

A									
Days	WBC (*10e9/L)	Neutrophils (*10e9/L)	Lymphocytes (*10e9/L)	Monocytes (*10e9/L)	Eosinophils (*10e9/L)	Basophils (*10e9/L)			
7	22.85	7.77	8.91	5.94 (26 %)	0.23	0			
23	13.69	5.75	2.87	6.16 (45 %)	0	0			
213	15.91	4.61	5.73	5.25 (33 %)	0.32	0			
Physiological mean (% of WBC)	15.51	3.77 (25.5 %)	5.02 (23.65 %)	6.46 (50 %)	0.24 (1.55 %)	0.02 (0.10 %)			
Days	RBC (*10e12/L)	HGB (g/dL)	HCT (%)	MCV (fL)	MCH (pg)	MCHV (g/dL)	RDW (%)	PLT (*10e9/L)	PCV (%)
7	3.54	13.4	37.7	107	37.8	35.5	14.3	**470**	36
23	5.06	19.2	55.1	109	37.9	34.8	14.5	968	55
213	3.62	13.3	39.2	108	36.8	33.9	13.9	996	37
Physiological (mean)	3.55	14.28	40.60	114.25	40.19	35.17	14.33	617.65	40.80
**B**									
Days	EEHV-1A (vgc/ml)	WBC (*10e9/L)	Neutrophils (*10e9/L)	Lymphocytes (*10e9/L)	Monocytes (*10e9/L)	Eosinophils (*10e9/L)	Basophils (*10e9/L)		
-33	5.0 × 10^2^	**23.08**	4.62	**5.77**	**12.46 (54 %)**	0	0		
-29	ND	**25.34**	4.56	**7.6**	**13.18 (52) %**	0	0		
-5	1.2 × 10^5^	**6.18**	3.46	**1.85**	**0.8 (13 %)**	0.06	0		
-3	2.7 × 10^6^	**7.35**	5.22	**1.32**	**0.81 (11 %)**	0	0		
0	7.2 × 10^6^	**13.25**	8.22	**2.65**	**2.25 (17 %)**	0.13	0		
1	1.9 × 10^7^	**12.925**	7.565	**2**	**2.975 (23 %)**	0	0.195		
Physiological mean (% of WBC)		15.51	3.77 (25.5 %)	5.02 (23.65 %)	6.46 (50 %)	0.24 (1.55 %)	0.02 (0.10 %)		
Days	RBC (*10e12/L)	HGB (g/dL)	HCT (%)	MCV (fL)	MCH (pg)	MCHV (g/dL)	RDW (%)	PLT (*10e9/L)	PCV (%)
-33	3.65	14.6	43.2	118.3	39.9	33.7	13.9	**950**	42
-29	3.58	14.5	41.4	115.5	40.4	35	38.1	**760**	38
-5	3.48	14.2	41.8	120.4	41	34	14	**560**	37
-3	3.36	14.2	38.4	114.4	42.4	37.1	13.7	**183**	38
0	2.89	12.4	32.9	113.7	42.9	37.8	13.8	**98**	34
1	3.07	12.95	35.05	114.2	42.2	36.9	13.6	**13**	36.5
Physiological mean	3.55	14.28	40.60	114.25	40.19	35.17	14.33	617.65	40.80

The haematological parameters for Case 1 (A) were only available for Days 7, 23 and 213 following suspicion of clinical EEHV-1A infection. The haematological parameters and viral load for Case 2 (B) were measured on several occasions from 33 days prior to the observation of signs indicative of clinical EEHV-1A infection (Day 0) to the Day 1 when the elephant succumbed to the infection at midnight. Normal platelet range of live juveniles in the collection was established, during a period of seven months from 20 blood samples of the elephants, to be at 372-878*10e9/L (mean of 625*10e9/L). The rest of the physiological parameters were from 24 measurements from four individuals under eight years of age (age range at time of sampling: 1.5-7y). Parameter values with a substantial change are highlighted in bold. Percentages of monocytes in the white blood cells are also given in the table

vgc/ml, viral genome copies per millilitre of EDTA whole blood; WBC, white blood cell count; RBC, red blood cell count; HGB, haemoglobin; HCT, haematocrit; MCV, mean cell volume; MCH, mean corpuscular haemoglobin; MCHV, mean corpuscular haemoglobin volume; RDW, red blood cell distribution width; PLT, platelet count; PCV, packed cell volume; ND, not detected

As a pre-emptive measure, the calf’s two siblings, a 3-year old male and a 4-year old female, were treated simultaneously with 12 mg/kg FCV TID from Day 0 to Day 6, which was tapered to 8 mg/kg BID from Day 7 to Day 12, after which treatment ceased. Neither sibling was positive for EEHV-1 DNA in blood or bodily secretions throughout this period.

**Fig. 3:**
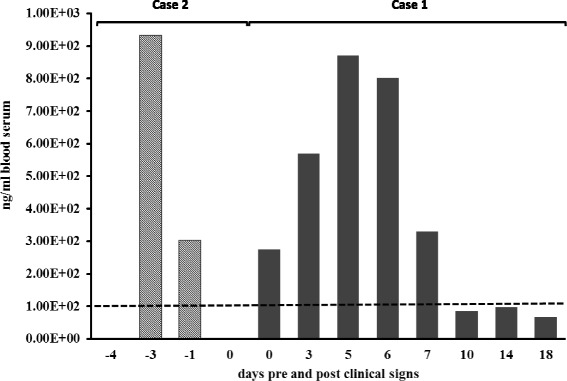
Penciclovir, the metabolite of famciclovir, plasma levels in 2 juvenile Asian elephants with EEHV-1A DNAemia. Famciclovir (FCV) was rectally administered in Case 1 at 15 mg/kg twice daily (BID) on observation of clinical signs indicative of EEHV-1A infection (Day 0) and continued at the same dose rate until Day 1, when it was reduced to 12 mg/kg thrice daily (TID). This was followed by further reduction on Day 3 to 8 mg/kg BID and continued unchanged until Day 18. In Case 2 FCV was administered at 15 mg/kg TID for two days following the detection of EEHV-1A DNA in the blood (Day -5), five days prior to observation of EEHV-1A clinical signs, and continued at the same dosage till the last dose of FCV at 9:00 am on Day 0; blood was only collected at 17:00 pm on that day. The death of Case 2 was at midnight on Day 1; seven days after first detection of EEHV-1 DNA in the blood. Dashed line indicates the minimum level of PCV considered therapeutic in humans with genital herpes (>100 ng/ml) [27]

Plasma PCV levels, measured at trough levels 8-10 hours following the latest FCV administrations in both Case 1 (Fig. 3) and its male sibling (data not shown), ranged between 68 and 934 ng/ml. Levels measured at the dosing regimens of 15 mg/kg TID and 12 mg/kg TID reached values considered therapeutic in humans with genital herpes (>100 ng/ml) [27].

### Case 2

In 2015, a blood swab collected during routine weekly sampling of 1.5 year old male calf ‘Max’ tested positive for EEHV-1 DNA by qPCR. This result was confirmed by a subsequent EDTA whole blood sample that tested qPCR positive at 5.0*10^2^ vgc/ml of blood. At this point monocytes were abundant (12.46*10^9^/L, 54 % of WBC) and the platelet count was 950*10^9^/L. (Table 1b, Day -33). A follow-up blood sample four days later was EEHV-1 DNA negative by qPCR and the calf remained negative on four subsequent blood swabs taken during a three-week period. On week 4 and one day prior to the calf being diagnosed again with EEHV-1 DNAemia the calf was presented with a swollen right temporal gland, which was painful on palpation and from which purulent discharge could be expressed. The gland was treated with pevidine (Sanofi, UK) flushes and topical amoxicillin/clavulanic acid application following bacteriological culture and sensitivity testing. After this positive EEHV qPCR result from a blood swab, an EDTA whole blood sample also tested positive, with a viral load of 1.2*10^5^ vgc/ml, the monocyte count had markedly reduced to 0.8*10^9^/L (13 % of WBC) and the platelet count almost halved to 560*10^9^ /L, in the absence of clinical signs (Day -5). PCR results for this sample became available two days after the actual sampling date and treatment was immediately instigated at 15 mg/kg FCV TID per rectum. Blood samples were then taken regularly for EEHV-1 qPCR, viral quantification and haematology.

Genomic analysis of this EEHV-1 by sequencing of PCR amplicons of partial viral POL, gL and gM genes identified the virus as EEHV-1A genotype. However, comparing these nucleotide sequences to those of Case 1, it was revealed that the virus was genomically slightly different, but both cases were more dissimilar to Riddle and Betts (Fig. 2). Nucleotide sequences of the partial segments of the three genes for Riddle and Betts were identical. This was not unexpected as the two fatalities occurred within two weeks of each other, but longer sequence reads would be necessary to verify this further.

Viral load increased exponentially as time progressed, during which the monocyte count stabilised around 2.54*10^9^/L (17 % of WBC), but platelet counts showed a continuous decline (Table 1b). Lymphocyte count has also shown substantial reduction on the detection of DNAemia, but other haematological parameters such as neutrophil and basophil counts remained unchanged. PCV plasma levels as high as 934 ng/ml were attained during the first days of treatment (Fig. 3). Five days into the DNAemia, the calf started to show signs of lethargy. At this point, PCV plasma levels had dropped to 303 ng/ml, about a third of its previous peak.

The decision was made to start i/v fluids, consisting of Harmann’s (15 ml/kg/h, Vetivex 11, Dechra, UK) supplemented with Duphalyte (1 ml/kg BW, Pfizer, UK), and complement the treatment from solely FCV per rectum to i/v plasma and GCV. The calf first displayed clinical signs of haemorrhagic disease six days into the DNAemia: oedema of the head and ventral abdomen, tongue petechiae and progressive signs of cyanosis. The platelet count had also reduced drastically at this point to 98*10^9^/L and viral load had increased to 7.2*10^6^ vgc/ml of blood. Intravenous cannulas were in place in both ears for administration of 1 ml/kg plasma TID, fluids and antibiotics (2 mg/kg marbofloxacin, Marbocyl®, Vetoquinol). Due to logistics, GCV (5 mg/ml) could not be administered until 16:00h that day, but achieved plasma levels of 2529 ng/ml following i/v injection. Clinical signs progressed despite treatment and the platelet count further reduced to 13*10^9^/L and viral load increased to 1.9*10^7^ vgc/ml of blood. The calf subsequently died at midnight, just into the next day as a result of hypoxaemia and hypovolaemia.

## Discussion

The early clinical signs of EEHV-HD are non-pathognomonic and often manifested as lethargy, anorexia and lameness. As a consequence of generalized capillary endothelial cell damage, these early clinical signs are likely to be followed by more severe and generally terminal signs [5, 8, 12]. Routine blood testing of at-risk Asian elephants for EEHV viraemia has been suggested as part of a monitoring scheme to predict and potentially prevent fatal disease, as the viral DNA can be detected at least several days prior to any clinical signs [13, 14]. This routine monitoring was proven to be valuable in this study, where Case 2 continuous EEHV-1 DNAemia was detected five days prior to the earliest clinical signs, although unfortunately this early initiated treatment was not able to prevent fatality.

In addition to detection and quantification of viral DNA through real-time qPCR, drop in the monocyte/neutrophil ratio, and absolute reduction of the platelet count, make both the monocytes and platelets the most useful prognostic haematological parameters of EEHV-HD, although the exact physiological process for these changes is as yet unknown. It is possible that the monocytes are subjected to immediate assault of the virus [19], and that the viral damage to the endothelial cells beyond the adaptive nature of the host response leads to the development of disseminated intravascular coagulation (DIC) and hence critical loss of platelets. In Case 2, the platelet count showed a gradual decline well before the fatal progressive viraemia, reaching a dangerously low level before death. Archived data from three previous EEHV fatalities at the same collection show a similar critically low platelet count following clinical EEHV-1 manifestation (Table 2). Similar findings have also been recently reported from Asian elephants affected by EEHV-1B, 4 and 5 viraemia [13, 18, 19]. It is noteworthy that mean haematological parameters vary substantially in Asian elephants’ blood, e.g. a reported range of 80-975*10^9^/L for platelets [31–35]. Monocytes are usually the most abundant white blood cells in the majority of juveniles. In these cases, the relative drop of monocytes, compared to neutrophils, in the early stages of the disease could easily be identified on straightforward in-house white blood cell differentials. There are, however, individuals in which the monocytes are on average less abundant and hence this early indicator will be less beneficial in these cases. It is therefore advisable to establish individual baselines for at least the abovementioned haematological parameters for each at-risk elephant in advance, and that clinicians familiarise themselves with in-house blood smear differentials.

**Table 2:** Haematological parameters of three additional fatal cases of EEHV-1A and EEHV-1B infections from the same zoological collection

Animals (date of sampling)	Virus type	WBC (*10e9/L)	Neutrophils (*10e9/L)	Lymphocytes (*10e9/L)	Monocytes (*10e9/L)	Eosinophils (*10e9/L)	Basophils (*10e9/L)		
Emilia									
(18/12/2006)	EEHV-1B	17	11.85	3.21	**1.9 (11.2 %)**	0.03	0.02		
Riddle									
(01/05/2009)	EEHV-1A	**2.71**	1.49	**0.84**	**0.27 (10 %)**	0	0		
Betts									
(15/05/2009)	EEHV-1A	**5.1**	3.11	**1.02**	**0.97 (19 %)**	0	0		
Physiological mean (% of WBC)		15.51	3.77 (25.5 %)	5.02 (23.65 %)	6.46 (50 %)	0.24 (1.55 %)	0.02 (0.10 %)		
Animals (date of sampling)	RBC (*10e12/L)	HGB (g/dL)	HCT (%)	MCV (fL)	MCH (pg)	MCHV (g/dL)	RDW (%)	PLT (*10e9/L)	PCV (%)
Emilia									
(18/12/2006)	3.05	12.8	39.2	122	42	34.4	15.7	**34.6**	NA
Riddle									
(01/05/2009)	3.55	13	37.7	106	36.7	34.6	17.4	**25.6**	35
Betts									
(15/05/2009)	3.16	12.9	55.1	112	40.8	36.5	17.3	**71.5**	NA
Physiological (mean)	3.55	14.28	40.60	114.25	40.19	35.17	14.33	617.65	40.80

Blood samples were taken following the observation of EEHV-1 infection clinical signs. WBC, lymphocyte, monocyte and platelet counts (highlighted in bold) consistently show substantial drop from their physiological level in the two fatalities caused by EEHV-1A. Percentages of monocytes in the white blood cells are also given in the table

WBC, white blood cell count; RBC, red blood cell count; HGB, haemoglobin; MCV, mean cell volume; MCH, mean corpuscular haemoglobin; MCHV, mean corpuscular haemoglobin volume; RDW, red blood cell distribution width; PLT, platelet count; PCV, packed cell volume; NA, not available

Monitoring kinetics of EEHV-1A load in the two elephants revealed rapid increase in viral load in the elephant that succumbed to the disease (Case 2) despite continuous anti-herpesviral treatment up to the EEHV-1 clinical signs. From at least three days prior to onset of clinical signs, PCV reached plasma levels suggested as therapeutic for treatment of diseases caused by alphaherpesviruses infections in humans [27], but here it had failed to control virus replication as the virus load continued to increase. Considering the approximate three hour half-life of PCV [27], the peak levels could have been up to three times higher than measured, as blood samples were taken 6-8 hours following the FCV administration.

The efficacy of FCV treatment to curb progression to EEHV-HD in clinical EEHV has always been debatable, as compromised cellular function at enteric level could affect absorption. This was indicated by the measured PCV level in Case 2, which dropped remarkably 48 hours prior to the onset of outwardly visible clinical signs. Factors such as drug expulsion and individual differences in drug metabolism are also considered possible causes of this drop [24]. These findings therefore favour those anti-herpesviral drugs that could be administered intravenously, to bypass the intestinal absorption step. Additionally, the available sequences for EEHV TK and PK have only 25 % amino acid identity with their orthologues in HSV and HCMV, target viruses for these drugs [9] and indirect surrogate in vitro assays have shown mild sensitivity for only the PK against PCV [36].

Taking into account the EEHV viraemia sequel following FCV administration in current and previous studies, the efficacy of FCV remains doubtful. Therefore the use of other, i/v administered and potentially more effective, anti- herpesviral therapies should be explored, especially in cases where pre-EEHV-HD viraemia, established through routine blood PCR monitoring, does not appear to be affected by FCV within the first days of treatment. In the absence of comprehensive data on a highly effective and safe anti-herpesviral drug, alternative drugs at present could include i/v administered nucleoside analogues such as GCV and ACV. The second-line drugs could be the pyrophosphate analogue foscarnet and the nucleotide analogue cidofovir, but these drugs all have their own safety issues. The most common adverse effect during GCV therapy in humans has been haematological toxicity, but this appears to be readily reversible on discontinuation of the drug [37]. It should also be noted that ACV is a hundred times more potent than FCV in inhibiting herpesviruses replication, but it suffers from a shorter half-life and intracellular concentration [38]. This drug has been recommended for clinical situations in humans where a rapid decrease in viral load is desirable, and could be of similar therapeutic value in EEHV clinical infections. The renal toxicity of the drug should also be considered in its therapy. Frequent measurement of EEHV load in blood samples is therefore of paramount value to establish the efficacy of ongoing therapy.

Complementary therapies that are likely to be of value include supportive therapies e.g. maintaining hydration and actions to prevent DIC and shock, such as i/v plasma administration from EEHV antibody positive adult donors, as a source of both antibodies and platelets, and a broad spectrum antibiotic to combat potential concurrent bacterial infections that might possibly be at the root of triggering the viral replication, predisposing to the infection or arise as a secondary infection.

Intravenous access appears to be of significant value for supportive treatment and anti-viral drug administration, and is straightforward in trained elephants that are used to being handled. The EEHV Advisory Group convened to debate this in 2014 and currently recommends minimum standards of care for all at-risk elephants irrespective of management systems, which include a section on Calf Training for intravenous access (www.eehvinfo.org). In elephants that have not attained this stage of training at the time of infection, repeated standing sedations using butorphanol and detomidine are possible, as used successfully in other institutions (Lauren Howard, personal communication).

As far as antibodies are concerned, Case 1 had been positive on an indirect ELISA for glycoprotein B (gB) about 9 months prior to the clinical signs and survived the viraemia [17]. On the other hand, Case 2 was also highly positive on the same gB ELISA (van den Doel, pers. communication) at the time of EEHV infection, but succumbed to the virus. For other closely related betaherpesvirus infections (e.g. HCMV), antibodies to the gB protein seemed to enhance protection by curbing at least the dissemination of the virus in the blood [29]. However, considering the fatality in Case 2 despite high anti-gB antibody, sole measurement of gB antibody may not be an indicator of protection against EEHV, as it is currently unknown which viral proteins are targeted by neutralising antibodies. The possibility also exists that early IgG antibodies to EEHV show a low avidity for the antigen and hence an avidity test is required to determine the value of antibodies against EEHV [39]. Also, in the view that herpesviruses (EEHVs and elephant gamma herpesviruses [EGHVs]) are ubiquitous in Asian elephant populations, the notion that the above mentioned ELISA is cross reactive with gB of similar viruses or other unknown agents cannot be excluded [9].

In this study, a detectable EEHV-1 DNAemia was correlated with the disease symptoms only when the blood viral load was at around 8.16*10^3^ vgc/ml of blood for Case 1 and 7.2*10^6^ vgc/ml for Case 2. A previous report states that a threshold of around 1.0*10^4^ vgc/ml of blood had coincided with apparent illness [14]. Clinical signs appeared at a much higher viral load in Case 2, emphasising a daily rapid increase in viral load as a more favourable determinant of initiating anti-herpesviral therapy, its success and disease prognosis. Our results also further endorse that DNAemia precedes virus shedding in trunk secretions of EEHV-1A infected animals, similar to those reported previously [13, 14, 18, 19]. Although there is no evidence that infectious particles are shed at a level sufficient to infect an in-contact animal, the potential risk these carrier elephants may pose following a clinical EEHV case should be considered.

## Conclusions

The EEHV viraemic cases investigated here further highlight the ongoing threat posed by the virus to the juvenile Asian elephants, potentially leading to gradual disappearance of their free-living population and an inability to maintain a viable captive population to safeguard the species from extinction. Therefore, effective control of future EEHV infections is fundamental to improve the survival rate of affected animals. Routine EEHV testing combined with viral quantification, WBC, monocyte and platelet counts following confirmed DNAemia in at-risk elephants should deliver an early warning of the clinical disease and prognosis, providing enough time to evaluate treatment opportunities to avert a fatal outcome. This investigation further questions the efficacy of the rectally administered FCV in controlling the viraemia and urges towards the exploration of alternative therapies. These therapies could include preferably i/v administered anti-herpes viral drugs, complementary plasma transfusions and antibiotics against potential secondary infections.

## Abbreviations

BID: Bis in die (twice a day)
DNA: Deoxyribonucleic acid
EDTA: Ethylenediaminetetraacetic acid
HPLC: High performance liquid chromatography
i/v: Intravenous
PBS: Phosphate buffered saline
PCR: Polymerase chain reaction
qPCR: Quantitative PCR
TID: Ter in die (thrice a day)
